# Marginalised Caregivers: A Qualitative Study of Grandmothers in Postpartum and Infant Care

**DOI:** 10.1002/nop2.70664

**Published:** 2026-07-19

**Authors:** Xiao Xiao, Xuanli Zhang, Ziyang Xie, Yunyi Zhang, Dadong Wu

**Affiliations:** ^1^ Outpatient and Emergency Department Shenzhen Maternity and Child Healthcare Hospital, Southern Medical University Shenzhen Guangdong China; ^2^ School of Nursing Southern Medical University Guangzhou Guangdong China; ^3^ Shenzhen Maternity and Child Healthcare Hospital, Southern Medical University Shenzhen Guangdong China

**Keywords:** child rearing, grandparents, intergenerational relations, postpartum period, qualitative research

## Abstract

**Aim:**

To explore grandmothers' experiences of sharing postpartum childcare responsibilities with their adult children as intergenerational co‐parenting relationships are formed and adjusted.

**Design:**

This is a descriptive qualitative study.

**Methods:**

Data were collected through face‐to‐face semi‐structured interviews from May to June 2019. A purposive sample was recruited from a tertiary maternal and child health hospital in Shenzhen, China. Data were analysed using inductive qualitative content analysis.

**Results:**

Sixteen grandmothers involved in postpartum and infant care were interviewed. Six themes were identified: (1) motivations for sharing parenting responsibilities; (2) navigating parenting differences; (3) roles of grandparents in childcare; (4) positive health impacts for grandmothers; (5) health challenges for grandmothers and (6) grandmothers' needs for parenting guidance.

**Conclusion:**

Grandmothers are active but often marginalised contributors to postpartum intergenerational co‐parenting. Their caregiving roles are shaped by family obligation, parenting disagreements, shifting family authority and unmet needs for professional guidance. Family‐centred postpartum interventions should include grandmothers as important members in the co‐parenting system.

**Implications for the Profession and/or Patient Care:**

In intergenerational co‐parenting families, grandmothers may experience both rewards and vulnerabilities as they provide postpartum and infant care. Nurses, midwives and community health professionals should recognise grandmothers as key caregiving partners and provide them with family‐centred guidance on intergenerational communication, role negotiation, infant‐care knowledge and caregiver well‐being.

**Patient or Public Contribution:**

Sixteen grandmothers shared their co‐parenting experiences through interviews and provided feedbacks on transcript summaries and preliminary interpretations.

**Reporting Method:**

This study was reported according to the SRQR and COREQ checklists.

**Impact (Addressing):**

- What problem did the study address?
  While grandmothers frequently provide care for postpartum mothers and infants in three‐generation households, their lived experiences, unmet needs and roles within co‐parenting dynamics remain understudied.
- What were the main findings?
  Grandmothers primarily provided care due to a sense of family obligation and reciprocity. They commonly acted as supporters and mediators, resolving parenting conflicts with their adult children. Additionally, they encountered both positive and adverse effects on their health, and voiced demands for accessible, practical and professionally delivered parenting support.
- Where and on whom will the research have an impact?
  This study yields meaningful impacts for multiple stakeholders and practice settings. The findings can guide postpartum nursing care, family‐centred maternal and child health services, and community health initiatives. They also offer evidence for developing targeted interventions for families with shared caregiving from grandparents.

## 1 Introduction

Intergenerational co‐parenting refers to the sharing of childcare responsibilities between parents and grandparents (Xiao and Loke 2022). Two broad patterns of intergenerational co‐parenting can be distinguished. In intact families, grandparents provide supplemental childcare support when parents need assistance. This arrangement has also been described as caregiving in three‐generation households, supplemental caregiving or non‐custodial caregiving (Guo et al. 2023). It is common in many Asian societies and in parts of Africa and Oceania (Chen et al. 2022; Kaluba et al. 2024; Matsui and Sato 2018). In recent years, such family structures have also become more common in some European countries and the United States, particularly in contexts of economic hardship, family crises or insufficient public support (Glaser et al. 2018). In non‐intact families, by contrast, grandparents may become the primary caregivers of grandchildren when parents are unable to provide care because of circumstances such as adolescent pregnancy, substance abuse, alcohol misuse or incarceration (Wang et al. 2019). Terms such as primary caregivers and custodial grandparents are often used to describe this arrangement (Wang et al. 2019). This family structure has more commonly been reported in regions such as the United States and some African countries (Wang et al. 2019; Bai et al. 2023).

Intergenerational co‐parenting is a common caregiving model worldwide. The 2018 China Health and Retirement Longitudinal Study showed that 46.39% of grandparents aged 45 years and above were involved in raising their grandchildren, with 59.52% of these cases occurring in three‐generation households (Chen et al. 2022). In Europe and the United States, fewer than 11.3% of adults aged 40 and older are raising their grandchildren; however, among these grandparent caregivers, at least 47% live in three‐generation households (Glaser et al. 2018). Despite the prevalence of grandparents' shared caregiving, previous family studies have focused mainly on custodial grandparents, with less attention to non‐custodial grandparents or three‐generation households (Danielsbacka et al. 2022).

## 2 Background

In China, economic development and the rising costs of childcare have increased the need for dual‐earner family arrangements to maintain household financial stability. Although public childcare services for children under the age of three have received renewed policy attention, the public childcare system remains underdeveloped (Xu and Bi 2022). As a result, grandparents have become an important source of childcare support for young parents.

Triadic interactions among grandparents, parents and grandchildren shape family dynamics and influence the psychological well‐being of all family members (Xiao and Loke 2022). Dyadic interactions between grandparents and parents shape intergenerational co‐parenting relationships, which in turn shape parenting practices (Chen et al. 2024). Such interactions may contribute to parenting stress, affect parent–child relationships and ultimately influence grandchildren's development (Zou et al. 2020). Grandparents' involvement in co‐parenting may have a dual effect. It can reduce parents' caregiving, financial and time burdens, but it may also trigger family conflicts, increase parents' parenting stress, and exacerbate grandparents' psychological distress (Xiao and Loke 2022). These conflicts may undermine the development of a nurturing family environment for children (Chen et al. 2024). Grandparents' participation may also have important short‐ and long‐term implications for children's developmental outcomes (He and Wang 2021). Despite grandparents' important role within the co‐parenting team, their experiences of co‐parenting with adult children and their understanding of family interactions within the family system remain insufficiently understood (Xiao and Loke 2022).

The postpartum period is a critical stage during which family members redefine their roles and establish a stable co‐parenting relationship (Xiao and Loke 2022). In Chinese families, it is common for grandmothers to care for postpartum women and newborns during this period (Xiao and Loke 2021a). In 2019, the State Council of China introduced measures to improve childcare services for children under 3 years of age, highlighting the need to strengthen support and guidance for family‐based infant care (Xu and Bi 2022). Against this background, this study explored grandmothers' experiences and perspectives regarding intergenerational co‐parenting during the postpartum period. The findings provide empirical evidence to inform the development of family‐focused interventions designed to improve co‐parenting experiences among grandmothers and their adult children.

## 3 Methods

### 3.1 Study Design

This study adopted a descriptive qualitative design guided by constructivism. Constructivism explores how participants construct meanings through social interactions and context, rather than assuming a single objective reality (Appleton and King 2002). This approach was utilised because the study sought to understand how grandmothers interpreted their caregiving roles, negotiated parenting responsibilities with adult children, and made sense of their position within postpartum co‐parenting relationships in Chinese families.

### 3.2 Study Setting

The study was conducted in a tertiary maternal and child health hospital in Shenzhen, China. Shenzhen is a highly urbanised migrant city in which dual‐earner families are common and many young parents rely on grandmothers for postpartum and infant care (Xiao and Loke 2021a). The hospital provides routine postnatal follow‐up at approximately 30 days, 42 days and 12 weeks postpartum, creating an appropriate setting for identifying families in which grandmothers were involved in caregiving.

### 3.3 Sampling and Recruitment

Grandmothers who were involved in intergenerational co‐parenting with a postpartum woman discharged from the study hospital between 20 May and 30 June 2019 were selected as potential participants. Grandmothers were eligible if they met the following criteria: (1) their adult children were married and registered as living in Shenzhen together with partners; (2) their daughter or daughter‐in‐law had experienced no complications during pregnancy or the puerperium; (3) their newborn grandchild was a healthy, full‐term singleton with a birth weight of at least 2500 g and (4) at least one grandmother participated in postpartum and infant care. Eligible grandmothers were required to be able to communicate in Mandarin and be willing to participate in an interview. Families were excluded if any family member had a psychological or mental illness.

Using a purposive sampling strategy, the target families were recruited from the postnatal women's health centre of the study hospital, where women returned for postpartum physical examinations at 1 month, 42 days and 3 months postpartum. Women were usually accompanied by their husbands or the infant's grandmother during these visits. The research team collaborated with nurses at the nursing station during recruitment. First, the purpose of the study was explained to postpartum women and grandmothers. Grandmothers who were present and expressed interest in participating were screened for eligibility. When eligible grandmothers were not present, postpartum women were asked to pass study information to the grandmothers or to give permission for the research team to contact them. In such cases, eligibility screening, informed consent and interview scheduling were conducted directly with the grandmothers. Only grandmothers who personally confirmed their caregiving involvement and willingness to participate were enrolled.

To obtain rich qualitative data, the team purposively sampled grandmothers with diverse backgrounds, including variations in kinship relationships, the number of adult children, educational background and household income. Each family received a shopping card worth 100 RMB after the interview.

Sample size was determined by data saturation. Saturation was assessed concurrently with data collection and preliminary analysis. After each interview, the research team reviewed transcripts, field notes and emerging codes. Saturation was considered to have been reached when subsequent interviews no longer generated new codes, categories or substantive variations. By the fourteenth interview, the coding framework had become stable. Two additional interviews were conducted to confirm saturation. These final interviews supported the existing themes and did not add new conceptual insights. Data collection therefore ended after 16 interviews.

The grandmothers were between 40 and 72 years old, with a mean age of 56.75 years (SD = 7.16). Eight (50.00%) grandmothers were maternal grandmothers, and another eight (50.00%) were paternal grandmothers. Eleven (68.75%) grandmothers had a middle school education or lower, four (25.00%) had completed high school and one (6.25%) had completed junior college. Six (37.50%) grandmothers had three or more adult children, five (31.25%) had two adult children and another five (31.25%) had one adult child (Table 1).

**TABLE 1:** Characteristics of grandmothers (*n* = 16).

ID	Age	Kinship	Education background	Household income (thousand CNY)	Number of adult children	Interview time (min)	Chronic conditions
P1	62	Maternal	High school	15,000	Three or more	35	No
P2	60	Maternal	High school	15,000	1	30	No
P3	72	Paternal	Middle school or lower	15,000	Three or more	45	Lumbar disc herniation
P4	50	Paternal	Middle school or lower	10,000	2	45	No
P5	61	Maternal	Middle school or lower	7500	1	35	No
P6	50	Maternal	Middle school or lower	10,000	2	35	Hypertension
P7	57	Paternal	High school	10,000	2	45	Headache
P8	55	Paternal	Middle school or lower	20,000	Three or more	52	Total hip arthroplasty
P9	57	Paternal	Middle school or lower	25,000	2	37	No
P10	56	Paternal	Middle school or lower	10,000	1	55	Joint pain
P11	58	Paternal	Middle school or lower	15,000	Three or more	30	Hyperlipidemia
P12	65	Paternal	Middle school or lower	5000	Three or more	45	Chronic cough, joint pain
P13	54	Maternal	Middle school or lower	5000	1	49	No
P14	52	Maternal	Middle school or lower	10,000	1	30	No
P15	40	Maternal	Junior college	7500	Three or more	44	Lumbar disc herniation
P16	59	Maternal	High school	12,500	2	45	Diabetes

*Note:* Household income refers to the average monthly disposable income of both parents (the average monthly per capita disposable income in Shenzhen in 2022 was 6059.85 CNY. [Statistics Bureau of Shenzhen Municipality, 2024]). CNY refers to the Chinese Yuan, the official currency of China.

### 3.4 Research Team and Reflexivity

The research team consisted of five female researchers with nursing backgrounds and experiences in perinatal care. The corresponding author was an obstetric nurse manager with PhD, MSN and RN credentials. A second senior researcher was a hospital‐affiliated associate researcher with a PhD degree. Both had training and experience in qualitative research design, data analysis, research ethics and maternal and child health research. The remaining three team members were Master students of Nursing who had received graduate‐level training in qualitative methods, transcription, coding and research ethics. All team members were proficient in Chinese and English. The corresponding author conducted all interviews.

The research team recognised that their nursing backgrounds and professional experience in maternal and child health could influence the formulation of interview questions, interpretation of data and interactions with participants. To enhance reflexivity, the researchers discussed their assumptions during team meetings, compared interpretations with the original transcripts and field notes, and remained attentive to meanings expressed in participants' own words.

### 3.5 Interview Outline

An interview outline was developed through discussions with several obstetric experts and a family nursing expert, based on the research objectives and a review of the literature. The interview focused on grandmothers' feelings, thoughts and concerns regarding their role in caring for the postpartum woman and newborn, as well as their interactions with adult children.

Initially, the interview question ‘Did you come here on your own, or did your child ask for your help with childcare?’ was used. After two pre‐interviews with grandmothers, this question was revised to ‘Why do you think you are involved in taking care of the mother and baby?’ to elicit richer accounts of grandmothers' motivations and meanings.

The final interview questions were: (1) How do you feel about being a grandmother? (2) Why do you think you are involved in taking care of the mother and baby? (3) Can you describe the process of caring for the mother and newborn? (4) How do you define your role in caring for the new mother and newborn? (5) Have you encountered any differing opinions from other family members when caring for the mother and newborn? If so, please give some examples. (6) How have you managed these conflicts, and how did they affect you and your family relationships? (7) Is there anything else you would like to talk about?

### 3.6 Data Collection

The interviewer had no prior personal relationship with the participants before recruitment and did not provide direct nursing care to them. Participants were informed that the interviewer was an obstetric nurse manager and the principal investigator of the research team. Initial rapport was established during recruitment and study introduction before the formal interviews. Participants were assured that their decision to participate or withdraw, as well as their interview responses, would not affect the healthcare services received by them or their family members.

The interview time and location were arranged in advance by telephone. Face‐to‐face semi‐structured interviews were usually conducted during home visits in a private room without other family members present. Before each interview, the researcher re‐explained the purpose, content and procedures of the study to the interviewee. Participants were assured that the interview material would remain confidential, would be accessible only to the research team and would not be disclosed to other family members.

After providing written informed consent, participants completed a sociodemographic information form. Each interview lasted approximately 30–55 min and was audio‐recorded. Field notes were taken to document participants' non‐verbal expressions and relevant contextual information about the interview setting. No repeat interviews were conducted. After transcription, transcript summaries and preliminary interpretations were returned to participants for comment and correction to confirm the accuracy of the interview content. No participant requested major changes to the transcripts or interpretations.

### 3.7 Data Analysis

Each audio‐recorded interview was transcribed verbatim by the interviewer within a week. Transcripts and field notes were imported into Excel and assigned anonymous codes. The file was accessible only to the members of the research team and was regularly backed up during the analysis. The transcription and data management processes were checked by two researchers.

Inductive qualitative content analysis was used to identify themes from the interview data (Kyngäs et al. 2020). The analysis followed an iterative process. First, all transcripts and field notes were read repeatedly to achieve immersion in the data. This helped the researchers obtain an overall understanding of grandmothers' postpartum caregiving experiences. Second, meaning units related to postpartum caregiving, infant care, intergenerational co‐parenting, role negotiation, parenting disagreements, health impacts and support needs were identified. These meaning units were condensed while preserving their original meanings. Third, condensed meaning units were labelled with open codes that stayed close to participants' own words. Similar codes were compared and grouped into sub‐categories according to similarities and differences in content. These sub‐categories were then further abstracted into sub‐themes and overarching themes.

Coding and theme development were conducted through iterative team discussions. Initial codes, sub‐categories, sub‐themes and themes were compared with the original transcripts and field notes to ensure that interpretations remained grounded in the data. Disagreements about coding, category boundaries or theme names were discussed among the research team until consensus was reached. The team moved back and forth between the transcripts, codes, sub‐themes and themes until the final thematic structure was agreed upon.

### 3.8 Ethical Considerations

This study was approved by the Research Ethics Committee of the study hospital (Approval no. SFYLS [2019]23) on 16 May 2019. Written informed consent was obtained from all participants.

### 3.9 Methodological Rigour

The methodological rigour of this study was enhanced using the criteria of credibility, dependability, confirmability and transferability. Credibility was supported through purposive sampling, rapport building, audio‐recording, verbatim transcription, field notes, member checking and representative quotations. The interviewer maintained a neutral attitude, avoided leading questions and used probing and clarification to verify participants' meanings. Dependability was strengthened by following a semi‐structured interview guide, documenting recruitment, data collection, transcription, coding and team discussions, and conducting data collection and analysis concurrently. Confirmability was enhanced through reflexivity, team discussion and repeated comparison of codes, sub‐themes and themes with transcripts and field notes. The interviewer did not provide direct nursing care to participants and reflected on her professional background in perinatal care to reduce potential bias. Transferability was addressed by describing the study setting, participant characteristics, recruitment procedures and sociocultural context. These descriptions enable readers to assess the applicability of the findings to similar postpartum co‐parenting contexts. To support translation accuracy, Chinese quotations were translated into English by the research team, reviewed by the corresponding author and edited by a native English speaker.

## 4 Results

Of the 28 families recruited, 16 grandmothers completed interviews (Table 1). The remaining 12 grandmothers withdrew before the formal interview because of time constraints or without providing a specific reason. Six themes and 16 sub‐themes were identified from the data (Table 2).

**TABLE 2:** Extracted themes and sub‐themes.

Themes	Sub‐themes	Number of participants contributing to each sub‐theme (*n*/*N*)	Participant IDs
1. Motivations for sharing parenting responsibilities	(1) Perceived family obligations	11/16	P1, P2, P3, P4, P6, P7, P9, P11, P14, P15, P16
	(2) Principle of reciprocity	1/16	P8
2. Navigating parenting differences	(1) Differences in parenting beliefs	4/16	P1, P3, P5, P9
	(2) Strategies for resolving disagreements	11/16	P1, P2, P4, P5, P6, P7, P11, P13, P14, P15, P16
3. Roles of grandparents in childcare	(1) Offering supportive childcare	9/16	P1, P2, P3, P4, P7, P8, P9, P12, P15
	(2) Mediating family dynamics	1/16	P15
4. Positive health impacts for grandmothers	(1) Becoming a happy grandmother	8/16	P1, P3, P4, P7, P9, P14, P15, P16
	(2) Receiving peer support	5/16	P3, P6, P8, P9, P15
	(3) Physical exercise through childcare	1/16	P10
5. Health challenges for grandmothers	(1) Interpersonal relationship stress	6/16	P1, P6, P7, P8, P10, P11
	(2) Social isolation	7/16	P1, P2, P6, P7, P9, P10, P11
	(3) Physical strain from childcare tasks	5/16	P1, P3, P7, P10, P16
	(4) Difficult postpartum adaptation period	2/16	P6, P10
6. Grandmothers' need for parenting guidance	(1) Acceptance of health education	9/16	P2, P3, P4, P7, P9, P10, P13, P15, P16
	(2) Preferences for learning methods	6/16	P3, P6, P7, P8, P9, P16
	(3) Inadequacies of current parenting guidance services	5/16	P1, P3, P5, P10, P13

### 4.1 Motivations for Sharing Parenting Responsibilities

Most grandmothers described caregiving as a family obligation (*n* = 11/16), while one explicitly framed her involvement in terms of intergenerational reciprocity. Their accounts suggested that postpartum and infant care were understood not only as practical support for adult children, but also as part of maintaining family continuity and mutual support across generations.

#### 4.1.1 Perceived Family Obligations

Participants' narratives showed that many grandmothers regarded helping their adult children as a natural extension of parental responsibility. They viewed themselves as older family members who were expected to help maintain household functioning and family harmony. Parental love also motivated them to become a caregiving resource for their adult children and to reduce the pressures faced by the younger generation.

(Haha) I had this idea to come here and take care of my granddaughter by myself. The nature of being a parent tells me that we need to give them some help when they need us even though they are adults now. (P1, maternal grandmother)

Although I could not provide financial support to my son, I would try my best to reduce his economic burden by giving him a hand in childrearing. As I am here to help, my son does not need to hire a nanny. (P4, paternal grandmother)

Caring for a newborn required substantial energy. Maternal and paternal grandmothers sometimes played interchangeable roles in sharing infant care responsibilities. When grandparents on one side of the family were unable to provide care because of poor health, geographical distance or other family obligations, grandparents on the other side often stepped in to provide substitute support.

My son‐in‐law's mother is over 70 years old. She can't take care of my granddaughter. I am a little younger than her, so I am here to help. (P1, maternal grandmother)

#### 4.1.2 Principle of Reciprocity

One motivation for assuming child‐rearing responsibilities was rooted in the expectation of long‐term reciprocity. While they remained physically able to provide care, some grandmothers helped their adult children in anticipation of receiving support later in life.

I'm afraid that if my son and daughter‐in‐law told me that they did not need my help to take care of my grandchildren, they might not be willing to support me when I am old. I will have nobody to rely on when I need it. (P8, paternal grandmother)

### 4.2 Navigating Parenting Differences

Although only a few grandmothers explicitly described parenting differences with adult children (*n* = 4/16), most accounts showed that grandmothers used various communication strategies to manage intergenerational disagreements about infant care (*n* = 11/16). These approaches appeared to be shaped by differences in family authority, educational background and individual communication preferences.

#### 4.2.1 Differences in Parenting Ideologies

Some grandmothers perceived a gap between their previous childcare experience and the infant‐care knowledge used by younger parents. This gap made them feel that traditional parenting practices were increasingly viewed as outdated, while younger parents had greater access to modern parenting knowledge via smartphones and other media. This shift reduced some grandmothers' perceived authority in infant care and made them more dependent on the guidance of younger family members.

Our experience primarily comes from the older generation in rural areas. In contrast, younger mothers often learn scientific parenting knowledge through their smartphones, and we do not have as much experience as they do. (P1, maternal grandmother)

#### 4.2.2 Strategies for Resolving Disagreements

Grandmothers addressed parenting differences through various communication strategies. Positive communication included listening, seeking consensus, consulting evidence and offering suggestions. Negative strategies included outward agreement combined with covert opposition.

Some grandmothers fully supported the parents' approach. They recognised that parents held primary responsibility and decision‐making authority and believed that scientific parenting might benefit the grandchild more than traditional methods.

Just listen to them (adult children) and do what they say. Young people can make their own decisions, but we still go with their ideas. After all, everyone just wants what's best for the grandchild. We only know the parenting methods from the past and don't really understand the modern scientific approaches. So, when the grandchild's mother is guiding us, we naturally follow her lead. (P4, paternal grandmother)

In some families, grandmothers preferred open dialogue and negotiation with parents to reach consensus on infant care practices.

We should respect the younger generation, allow them to express their opinions and listen to their reasonable advice. Then there would be a smaller generation gap between us. (P6, maternal grandmother)

Some grandmothers maintained clear generational boundaries with their adult children. They upheld their own views but avoided interfering in infant care decisions. When consensus could not be reached, they sometimes chose to compromise to reduce family conflict.

I just offered my opinions when I was asked to. I try to respect her choice and not interfere too much. (P14, maternal grandmother)

Grandmothers with higher education levels sometimes adopted a more evidence‐based approach by consulting authoritative resources to resolve parenting disputes.

When there were different opinions between me and the parents of the child, we would not argue about who was right or wrong. We would search for evidence or authority to decide the best way instead. (P16, maternal grandmother)

When agreement could not be reached and grandmothers were unwilling to compromise, they sometimes implemented their own methods without the parents' knowledge, which could create hidden tension within the family.

One time, the baby was crying, and I suggested giving some water, but the mom said the doctor recommended exclusive breastfeeding, so water wasn't needed. I figured, ‘Well, adults drink water after meals.’ So when the mom wasn't around, the paternal grandparents and I secretly gave the baby some water together. (P13, maternal grandmother)

### 4.3 Roles of Grandmothers in Childcare

Grandmothers most commonly positioned themselves as supportive caregivers who assisted with childcare and household tasks (*n* = 9/16). Among them, one participant also described taking on a mediating role in family conflicts. Their accounts suggested that they often supported, rather than directed, postpartum and infant care decisions.

#### 4.3.1 Offering Supportive Childcare

As mothers recovered during the postpartum period and fathers engaged in paid work to support the family financially, grandmothers assumed a substantial share of household responsibilities. However, because some grandmothers felt they lacked up‐to‐date scientific parenting knowledge, they did not always provide direct care to the newborn. In families with more than one child, grandmothers often cared primarily for older grandchildren or undertook household tasks.

I take charge of cleaning, cooking, and washing clothes for the family. My husband's task is to take the older grandchildren to and from kindergarten. (P3, paternal grandmother)

My daughter‐in‐law takes care of my younger granddaughter every day, and I take care of the older one with my husband. (P9, paternal grandmother)

#### 4.3.2 Mediating Family Dynamics

The birth of a new baby sometimes intensified couple conflicts as parents adapted to their new roles. Some grandmothers acted as mediators, helping to reduce tension and maintain family harmony.

I always criticise my daughter when she and her husband quarrel with each other. I think my daughter sometimes talks too much. I will stand in the middle and act as a judge to mediate. I should be fair as a parent. Now their relationship has improved a lot. (P15, maternal grandmother)

### 4.4 Positive Health Impacts for Grandmothers

Grandmothers reported several positive experiences associated with caregiving, including emotional rewards from grandchildren (*n* = 8/16), peer support from neighbours or nannies (*n* = 5/16) and perceived physical benefits from caregiving‐related activity (*n* = 1/16).

#### 4.4.1 Becoming a Happy Grandmother

Some grandmothers described emotional satisfaction and a sense of fulfilment from interacting with their grandchildren. These positive experiences appeared to strengthen their attachment to the grandchild and their sense of belonging within the family.

I enjoy spending time with my grandson as he grows up. The child looks so small, but he responds with facial expressions when you make a sound or talk to him. When I see his smile, I feel interested and entertained. (P15, maternal grandmother)

#### 4.4.2 Receiving Peer Support

Grandmothers noted that caring for postpartum women and newborns was demanding and also involved a learning process. Peer support from nannies and neighbours was therefore valuable during this period.

The nanny we hired helped us a lot. Thanks to her, I didn't feel too tired. (P3, paternal grandmother)

When I take the baby out, it's nice to chat with the neighbours about parenting tips and learn from each other. (P15, maternal grandmother)

#### 4.4.3 Physical Exercise Through Childcare

When caregiving tasks were within their physical capacity, some grandmothers viewed caregiving‐related labour as a form of exercise. In this case, participation in childcare was perceived as beneficial to physical health.

I didn't do much exercise before. Since taking care of my grandson, I have been mainly responsible for the housework, and the amount of labour has increased. Parenting is also a form of exercise for me. (P10, paternal grandmother)

### 4.5 Health Challenges for Grandmothers

Most grandmothers did not explicitly describe caregiving as causing illness or psychological distress. However, their narratives revealed several caregiving‐related challenges, including social isolation (*n* = 7/16), interpersonal relationship stress (*n* = 6/16), physical strain (*n* = 5/16) and difficulty adapting during the early postpartum period (*n* = 2/16).

#### 4.5.1 Interpersonal Relationship Stress

Some paternal grandmothers described tension in their relationships with daughters‐in‐law, particularly when they felt blamed or insufficiently respected during infant care.

One time, I caught a cold and might have passed it on to my grandson, and my daughter‐in‐law blamed me for it. Honestly, taking care of a kid is pretty stressful—I'm always worried something might go wrong. (P10, paternal grandmother)

When grandparents had more than one adult child, some grandmothers had to live separately from their husbands. Each spouse stayed in a different adult child's household to share childcare responsibilities across families. Prolonged separation could limit a grandmother's ability to fulfil responsibilities towards her spouse and might affect the marital relationship.

I have three sons, each with two babies. My husband has to stay in our hometown to look after our second son's child. I have to stay here to take care of the younger son's baby. (P11, paternal grandmother)

#### 4.5.2 Social Isolation

Several grandmothers described experiences of social isolation after moving to their adult children's households to provide postpartum and infant care. For some participants, relocation to a new urban environment disrupted their previous social networks and made it difficult to participate in familiar leisure activities. Differences in local customs, recreational activities and social routines made them feel disconnected from older adults in the new community. In addition, caregiving and household responsibilities limited the time and energy available for social interaction. Some grandmothers reported having little opportunity or motivation to engage in activities outside the home. Although some participants interacted with neighbours or peers while taking the baby outside, these interactions did not necessarily involve discussion of family difficulties or emotional support. As a result, social interactions were often limited and did not always translate into meaningful peer support.

The leisure activities older people enjoy here are different from those in my hometown. For example, the rules of Mahjong and the dancing styles are different from ours. I find myself left out of the group. (P6, maternal grandmother)

I have to look after my grandchild. I have neither the time nor the desire to go out dancing or singing. (P7, paternal grandmother)

When I take the baby out, I do chat with other people my age, but I'm not really interested in talking about my own family, and I'm not interested in other people's family matters either. (P7, paternal grandmother)

#### 4.5.3 Physical Strain From Childcare Tasks

Among the interviewed grandmothers, 15 were over 50 years of age and five were over 60. Nine reported having chronic illnesses. These grandmothers indicated that caring for a newborn during the postpartum period placed considerable demands on their physical strength and energy. Physical fatigue and sleep deprivation were common concerns.

I have had hip injuries before. My hip joint swells up when I walk too much. I feel much better when I sit down. When I hold the baby, I walk slowly. I'm afraid of falling with the baby in my arms. The doctors told me not to break my hip again. (P8, paternal grandmother)

It's relatively tiring to take care of a new mother and newborn during the postpartum period. I can't sleep well at night since I have to be there when the new mother and the newborn call me. (P16, maternal grandmother)

#### 4.5.4 Difficult Postpartum Adaptation Period

The first 3 months postpartum are a crucial period for establishing a stable co‐parenting relationship within the family (Xiao and Loke 2022). During this period, family members must adjust to the newborn's temperament and daily routine, which may cause initial difficulties. As this adjustment phase progresses, family members may become more confident in their roles and caregiving routines.

We didn't know why the baby was crying. A crying baby made it impossible for the whole family to rest and caused everyone to feel exhausted. Then everyone was irritated. I think the main reason was that none of us were childcare experts, so it was easy for us to feel nervous and anxious. But now the baby is two months old, and we can read his signals. (P10, paternal grandmother)

### 4.6 Grandmothers' Need for Parenting Guidance

Grandmothers' accounts showed a clear need for parenting guidance. More than half expressed willingness to receive health education (*n* = 9/16), while several described preferred ways of learning infant‐care knowledge (*n* = 6/16) and suggested improvements to current hospital‐ or community‐based services (*n* = 5/16).

#### 4.6.1 Acceptance of Health Education

More than half of the grandmothers explicitly expressed a willingness to participate in parenting guidance services, such as maternity schools or online courses. Only one grandmother stated that her personal experience was sufficient for caregiving tasks and declined to learn new parenting knowledge. Many grandmothers acknowledged that their previous parenting experience had either been forgotten or become outdated and was therefore insufficient for current infant care. They expressed a desire to learn scientific maternal and infant care knowledge to participate more effectively in newborn care.

If time permits, it is necessary to attend some courses on how to take care of a baby and how to manage the family. In that case, I would know how to deal with problems when I encounter them. (P9, paternal grandmother)

I've already raised two kids myself, so there's no need to learn anything new. I don't need to go to the hospital (for maternity classes), or take online courses either. (P15, maternal grandmother)

#### 4.6.2 Preferences for Learning Methods

Grandmothers acquired parenting knowledge from nannies, the older generation and adult children. They also used books, videos and the internet to access information. When families encountered challenges such as infant discomfort or maternal breast engorgement, they could become vulnerable to misleading advertisements and ineffective expenditures. Participants tended to regard healthcare professionals as trusted sources of information because family members recognised their professional expertise in maternal and infant care.

A lot of things I didn't know how to do. I usually learned most of it by watching healthcare professionals, reading books, watching videos, or using Baidu. (P16, maternal grandmother)

My daughter's breasts were swollen in the first days after childbirth. We hired two lactation consultants, each costing 1,000 yuan, to help get the milk flowing, but it didn't work well, and the breast was still engorged and hard. The nanny suggested covering the breasts with potato slices, while the nurses told her that the cabbage leaves were more helpful. In the end, the cabbage leaves worked. (P13, maternal grandmother)

#### 4.6.3 Inadequacies of Current Parenting Guidance Services

Grandmothers found it difficult to attend existing prenatal classes because of fixed schedules and distant locations. They also perceived the content as monotonous, insufficiently personalised and mainly delivered through lectures and videos. Participants preferred guidance that combined theoretical explanation with practical demonstration. Some participants perceived health education in postpartum wards as inadequate and of limited benefit to families. Additionally, the quality of post‐discharge community home‐visit services varied by region, and some areas had lower visit frequencies.

Where my daughter lives, the community nurse used to visit her once a week after childbirth. Here, it is now once every two weeks (voice rising). I still wish they could give us more guidance. (P1, maternal grandmother)

The teaching methods in the hospital's prenatal classes are pretty simple. They mostly show foreign videos and rarely share any postpartum care experiences. (P3, paternal grandmother)

I feel like the doctors and nurses didn't really guide the new moms on what to watch out for. They were supposed to teach us, but they didn't. (P10, paternal grandmother)

## 5 Discussion

**FIGURE 1:**
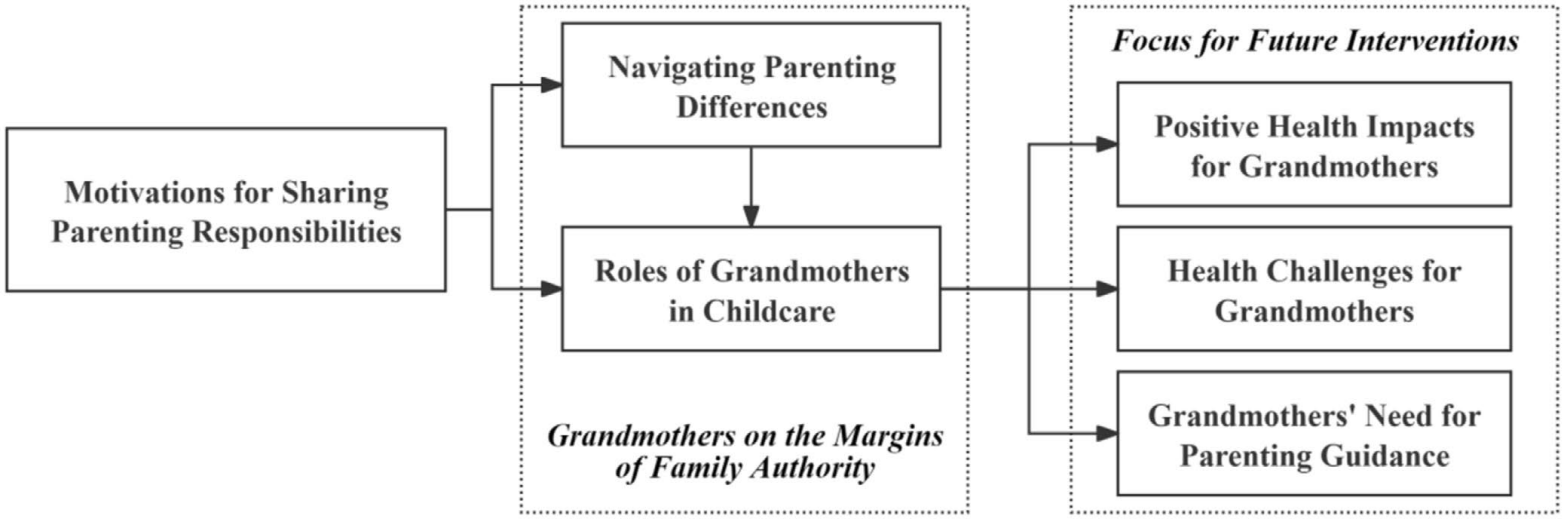
Relationships of the findings themes.

The themes identified in this study showed several internal connections (Figure 1). Motivated by grandparental responsibility and expectations of care in later years, grandmothers shared the work of raising grandchildren with their adult children. However, differences in parenting beliefs between generations often required grandmothers to adopt communication strategies to maintain family harmony. Their childcare roles were shaped by these beliefs and family authority dynamics, often positioning them as peripheral supporters rather than primary decision‐makers. The positive and negative impacts of caregiving interacted in ways that could influence grandmothers' overall well‐being. Although grandmothers trusted healthcare professionals, existing family‐based parenting guidance did not fully meet their needs.

### 5.1 Motivation for Sharing Childcare Responsibilities

Consistent with previous studies, grandmothers in this study were motivated to share parenting responsibilities with their adult children because of family obligations (Hoang and Kirby 2020; Shorey and Ng 2022). This finding may be interpreted through the theory of intergenerational solidarity (Duflos and Giraudeau 2021). In China, grandmothers' involvement in childcare is often regarded as a normative duty, embedded in cultural expectations and familial obligations, reflecting normative solidarity. Caring for grandchildren is also a way for grandmothers to express love for adult children and demonstrate affective solidarity. Furthermore, grandmothers assume caregiving responsibilities partly because of insufficient external childcare resources, illustrating structural solidarity. Finally, grandmothers enact functional solidarity by helping with childcare to ease adult children's burdens. Their caregiving may also reflect the hope of receiving support in later years and maximising family welfare across generations.

These motivations suggest that grandmothers are likely to remain involved in childcare in the foreseeable future, making grandparents' roles in co‐parenting families an important area for future research. Previous research indicates that stronger willingness among migrant grandparents to raise grandchildren is associated with more positive self‐rated health (Wang et al. 2022). Grandmothers who voluntarily participate in childcare may experience fulfilment in their grandparental role, whereas those who participate involuntarily may experience adverse health effects (Chan et al. 2023). Healthcare professionals should therefore assess the degree of voluntariness of grandparents' participation in childcare and develop tailored guidance and support programmes.

### 5.2 Communication Strategies for Childcare Disagreements

This study found that grandmothers often disagreed with parents about childcare beliefs and practices. Conflicts between grandparents and parents primarily arise from differences in childcare practices (Hoang and Kirby 2020). In other studies, parents have managed these differences through negotiation or avoidance, often influenced by cultural expectations of filial piety (Shorey and Ng 2022). In the present study, however, grandmothers generally respected and adopted the parents' decisions in childcare. This may be because grandparents recognised the perceived benefits of the parents' scientific childcare methods, reflecting the neo‐familism value that prioritises the well‐being of grandchildren (Zhao and Huang 2018; Hoang and Kirby 2020).

The study also revealed that grandmothers mainly used positive communication strategies, such as compliance, discussion and suggestion‐giving. Even when their views were not accepted, they did not usually insist on imposing their opinions on parents. This approach indicates that grandparents valued clear family boundaries, conflict avoidance and consistency in childcare decisions (Xiao and Loke 2022; Hoang and Kirby 2020). Unlike traditional filial piety, which emphasises obedience from younger generations, this communication pattern reflects a new filial culture in which elders respect the autonomy of younger family members (Zhao and Huang 2018). Both neo‐familism values and this new filial culture indicate that authority within the family increasingly resides with parents, who often have higher economic status and educational attainment (Zhao and Huang 2018; Bai et al. 2023). In contrast, grandparents may lack autonomy and decision‐making power within the family (Zhao and Huang 2018; Bai et al. 2023).

However, limited authority may prevent grandparents from openly challenging parents' practices, potentially leading to long‐term psychological impacts through passive acceptance (Zhao and Huang 2018). Additionally, the interviews did not reveal any power struggles between grandmothers and parents, but this may partly reflect social desirability bias. Grandmothers also expressed strong trust in health professionals. Therefore, childcare guidance interventions should provide evidence‐based information to reduce intergenerational differences in childcare practices and help family members develop effective communication skills.

### 5.3 Family Power Structure and Parenting Roles

Grandparents' childcare roles are influenced by their authority within the family (Bai et al. 2023). This authority is shaped by the economic and cultural differences between grandparents and their adult children (Zhao and Huang 2018; Bai et al. 2023). A meta‐ethnography synthesis of co‐parenting in Asian families indicates that the parents' and grandparents' childcare roles often overlap, with grandparents frequently serving as educators and cultural transmitters (Hoang and Kirby 2020). In contrast, this study identified a relatively clear division of labour between grandmothers and parents. Parents acted as managers and primary decision‐makers, while grandmothers served as assistants and were guided by parents in the adoption of scientific childcare methods.

Because newborn care was perceived as high risk, parents often provided direct newborn care themselves or hired professional nannies, while grandparents were assigned to older‐child care or household chores. This division of labour reflects differences in authority between generations within the family (Bai et al. 2023). Similar to other studies, our findings suggest that grandparents often assume the roles of caregivers and mediators, undertaking physically demanding childcare tasks and helping to resolve family conflicts (Hoang and Kirby 2020; Xiao and Loke 2021a).

These findings indicate that the authority structure within the family should be considered when delivering family childcare guidance. Interventions may need to target family members with higher decision‐making authority while also ensuring that grandmothers receive direct support and opportunities to express their needs.

### 5.4 Grandparents' Health and Adaptation to Parenting Roles

Research on the health and well‐being of grandparents in multigenerational families has produced mixed findings. Systematic reviews on supplemental grandparenting and grandparental involvement in Asian families suggest potential physical and mental health benefits, but substantial heterogeneity exists across studies (Danielsbacka et al. 2022). In this study, grandmothers reported both benefits and challenges to their physical and mental health. These findings can be understood through role enhancement theory and role strain theory (Notter 2022). Role enhancement theory suggests that caring for grandchildren can alleviate loneliness, enhance self‐worth and provide emotional support from adult children, thereby helping grandparents adapt to and strengthen their caregiving role. Conversely, role strain theory suggests that when caregiving demands exceed grandparents' capabilities and resources, grandparents may experience stress and physical or mental discomfort that hinders their ability to fulfil the caregiving role.

Consistent with previous studies, some grandmothers reported fulfilment from enacting familial roles and meeting social expectations (Shorey and Ng 2022; Bai et al. 2023). They also developed emotional bonds through interactions with family members, which provided a sense of joy, belonging and achievement (Shorey and Ng 2022; Bai et al. 2023). However, grandmothers also experienced relationship tensions and conflicts, which could produce feelings of disrespect, powerlessness and loneliness. While some grandmothers believed that caregiving activities benefited their health, others reported chronic disease recurrence, physical fatigue and sleep deprivation.

Research indicates that grandparents who engage in low to moderate levels of caregiving are more likely to experience fewer mobility limitations, fewer depressive symptoms and better cognitive function (Zeng et al. 2021). It may therefore be advisable for grandparents to participate in childcare at a moderate level and to have their health monitored. Given the regional limitations of China's health insurance and community health services, future efforts should consider incorporating health monitoring of caregiving grandparents into community health programmes.

In terms of social support, grandmothers may receive assistance from neighbours, friends and nannies. Social support has been shown to have a positive effect on grandparents' subjective well‐being (Guo et al. 2023). However, this study also found that some grandmothers were at risk of social isolation. The demanding nature of childcare consumes much of their personal time, reducing opportunities to engage in enjoyable activities and limiting social interactions. Their reluctance to discuss family difficulties with outsiders may be understood in relation to cultural expectations of maintaining family privacy and harmony in Chinese families. Such beliefs make grandmothers less likely to discuss difficulties with others, especially when these difficulties involve conflicts with family members. Greater social participation has been shown to improve mental health (Notter 2022). Social isolation may therefore restrict grandmothers' access to support and contribute to negative psychological effects. Future interventions should consider how to establish a hospital–community–family support system for grandparents within this cultural context.

The interviewed grandmothers mentioned that the first 1–2 months after childbirth represented a challenging adjustment period as family members learned to manage newborn discomforts and develop caregiving routines. The first 3 months after delivery are crucial for the family to establish a stable and cooperative caregiving relationship and for parents and grandparents to transition smoothly into their new roles (Xiao and Loke 2022). Previous research indicates that co‐parenting interventions commonly last 6 weeks to 6 months and cover the postpartum period (Xiao and Loke 2021b). The postpartum stage is an optimal time for intervention because families often lack the caregiving knowledge and skills to manage maternal and infant discomforts. Although existing childcare interventions developed for grandparents may benefit family members, they often fail to meet grandparents' specific needs adequately (Sherr et al. 2018). Future research should develop family caregiving interventions tailored to grandparents' needs and assess intervention effects from grandparents' perspectives.

### 5.5 Postpartum Intervention Needs of Co‐Parenting Grandmothers

There remains a significant gap in understanding the organisational support needs of non‐custodial grandparents (Shorey and Ng 2022). This study explored grandmothers' needs in depth and found that many were willing to proactively learn new knowledge. This suggests that grandmothers not only held a positive attitude towards their caregiving roles but also wished to remain relevant and influential within the evolving family structure. Training and educational programmes tailored to grandmothers may therefore have substantial acceptability.

Grandmothers generally expressed trust in healthcare professionals and valued the authority of professional opinions. This trust provides an important foundation for healthcare professionals to play a critical role in designing and implementing interventions. By drawing on professional credibility and expertise, family interventions may communicate scientific childcare knowledge more effectively and help grandmothers manage caregiving challenges.

Despite this trust in healthcare professionals, existing support and education did not fully meet grandmothers' needs. Current health education content and delivery methods may not adequately address their individualised needs. Future efforts should develop flexible, personalised and diverse training programmes that combine theory with practice and are delivered through both online and offline formats. Health education in postpartum wards should be standardised, personalised and thoroughly implemented. In community health services, the lack of uniform standardisation may lead to inconsistent service quality. Future efforts should promote standardisation and equity in community health services and ensure that guidance for family‐based postpartum and infant care is consistently delivered.

## 6 Limitations

Several limitations should be acknowledged. Although all participants were pre‐screened, the sample may not fully represent all grandmothers involved in intergenerational co‐parenting because recruitment took place in a single health centre in an economically developed city. The withdrawal of 12 grandmothers before the formal interview may have introduced non‐response bias. Despite the interviewer encouraging grandmothers to express themselves freely and openly, the findings may have been influenced by the cultural norm of ‘avoiding discussion of family matters with outsiders’. Our study focused exclusively on grandmothers, so future research should also examine grandfathers' experiences in intergenerational co‐parenting families. Finally, because the study was conducted in Shenzhen, the findings may not be transferable to rural areas or regions with different socioeconomic and healthcare contexts.

## 7 Future Research

The themes identified in this study provide important insights into modifiable factors that may influence the health and well‐being of grandparents involved in intergenerational co‐parenting. Future research should include quantitative studies to examine factors associated with grandparents' health outcomes. These variables may include social support, physical activity, physical fatigue, chronic illness, intergenerational relationships, marital relationships and caregiving intensity.

In addition, future studies should develop and evaluate family‐centred intervention programmes tailored to the needs of grandmothers and other caregiving grandparents. Such programmes may focus on enhancing caregiving confidence, improving intergenerational communication, reducing caregiving burden and promoting grandparents' physical and psychological well‐being in postpartum and infant care contexts.

## 8 Conclusions

Chinese grandmothers strive to fulfil expected family roles and maintain family harmony during the postpartum period. However, as family authority increasingly shifts towards the younger generation, grandmothers' traditional parental authority may be weakened, influencing their caregiving roles and intergenerational communication. While supporting postpartum women and infants, grandmothers experience both rewards and sacrifices in relation to their physical and psychological well‐being.

Healthcare professionals should recognise grandmothers as important members of the postpartum co‐parenting system and pay close attention to their caregiving experiences, health needs and support preferences. Comprehensive, family‐centred guidance should be provided to help grandmothers manage co‐parenting, strengthen communication with adult children and safeguard their own well‐being. The findings may be useful for regions with similar cultural, family and economic contexts.

## Author Contributions

**Xiao Xiao:** conceptualisation, data curation, formal analysis, funding acquisition, investigation, methodology, project administration, supervision, validation, writing – original draft, writing – review and editing. **Xuanli Zhang:** formal analysis, methodology, validation, visualisation, writing – original draft, writing – review and editing. **Ziyang Xie:** formal analysis, methodology, validation, visualisation, writing – original draft, writing – review and editing. **Yunyi Zhang:** formal analysis, methodology, validation, visualisation, writing – original draft, writing – review and editing. **Dadong Wu:** formal analysis, methodology, validation, writing – review and editing.

## Funding

This work was supported by the Shenzhen Science and Technology Innovation Commission (JCYJ20210324125204012, JCYJ20230807120312026), the Nursing Special Project of Shenzhen Maternity and Child Healthcare Hospital (FYB2022003) and Sanming Project of Medicine in Shenzhen (SZSM202211032). Those funding organisations did not interfere with the survey's design, implementation and analysis.

## Ethics Statement

The Research Ethics Review Committee at the study hospital approved our interviews (approval: SFYLS [2019]23) on 16 May 2019. Respondents gave written consent for review and signature before starting interviews.

## Conflicts of Interest

The authors declare no conflicts of interest.

## Supporting information


**Table S1:** SRQR checklist.

**Table S2:** COREQ checklist.

## Data Availability

The data that support the findings of this study are available on request from the corresponding author. The data are not publicly available due to privacy or ethical restrictions.
